# Development of patient‐specific pre‐treatment verification procedure for FLASH proton therapy based on time resolved film dosimetry

**DOI:** 10.1002/mp.17534

**Published:** 2024-11-27

**Authors:** K. H. Spruijt, J. Godart, M. Rovituso, Y. Wang, E. van der Wal, S. J. M. Habraken, M. Hoogeman

**Affiliations:** ^1^ HollandPTC Delft The Netherlands; ^2^ Department of Radiotherapy Erasmus MC Cancer Institute University Medical Center Rotterdam Rotterdam The Netherlands; ^3^ Department of Radiation Oncology Leiden University Medical Center Leiden The Netherlands

**Keywords:** FLASH, Gafchromic film, pre‐treatment verification

## Abstract

**Background:**

Pre‐clinical studies demonstrate that delivering a high dose at a high dose rate result in less toxicity while maintaining tumor control, known as the FLASH effect. In proton therapy, clinical trials have started using 250 MeV transmission beams and more trials are foreseen. A novel aspect of FLASH treatments, compared to conventional radiotherapy, is the importance of dose rate next to dose and geometry. Therefore, to ensure the safety and quality of FLASH treatments, patient‐specific dose‐rate verification before treatment is an important additional prerequisite. Various definitions of dose rate have been reported, however, the scanning proton beam (PBS) dose rate definition of Folkerts 2020 is currently the most used. It is the ratio between delta dose (ΔD) and delta time (Δt), subject to a predefined threshold, for a given position. Gafchromic film is a widely available detector used to perform relative and absolute integrated dose measurements. Since the response time of film is in the order of micro seconds it could also be suitable for pre‐treatment verification of FLASH proton therapy.

**Purpose:**

Development of a patient‐specific pre‐treatment verification procedure for FLASH proton therapy based on time resolved film dosimetry. The detector design is presented and validated using three tests.

**Methods:**

A dedicated setup was built that holds a Gafchromic film and a high‐speed camera to record the film during the irradiations. The red color channel of the camera's readings was converted into optical density (OD) and an OD‐to‐dose calibration curve was applied to determine the relative dose accumulation over time. To undo the film measurement (film response) of the post‐irradiation coloration process, it is assumed that each dose deposit (pulse) results in a similar film response function. The convolution of the film response function over the pulse provides the film response. First the film response function was obtained by fitting this parameter onto a known film response and corresponding pulse. Post‐irradiation coloration correction was performed by deconvoluting all film measurement by the obtained film response function. From the integral of each measured pulse, the Δt was obtained. Several validation tests were conducted: compare the Δt film measurement to a reference detector, exclude that revisiting spots result in an unwanted artefact on the dose accumulation measurement and thereby Δt, and compare Δt distributions of film measurement and simulation (local gamma evaluation, criteria 10%/2 mm) for nine QA fields (dose values; 12, 15, and 20 Gy, and, nozzle currents; 25, 120, and 215 nA). A similar analysis was performed for three dose optimized treatment beams, with and without scan patterns optimized on local dose rate.

**Results:**

Good agreement was found for Δt comparing film to the reference detector, but for Δt values smaller than ∼20 ms the error becomes larger (≥15%). Dose accumulation measured with film over time from a single spot is independent of whether the dose is delivered at once, twice or thrice. All gamma evaluations resulted in a gamma pass rate of ≥90%.

**Conclusions:**

Time resolved film dosimetry to perform patient‐specific pre‐treatment verification in FLASH proton therapy is feasible.

## 1 INTRODUCTION

Pre‐clinical studies have shown that applying ultra‐high dose rate (UHDR) irradiation, combined with high dose per fraction, result in additional healthy‐tissue spearing while maintaining tumor control.^1^, ^2^, ^3^ The beneficial effect was termed the FLASH effect, typically reported values for dose rate and dose thresholds are 40–100 Gy/s and 3.5–7 Gy, respectively.^4^ The first clinical trial to proof the feasibility of UHDR proton therapy applying 250 MeV proton transmission beams has been completed.^5^ A follow‐up study to asses pain relief and toxicity after treatment of symptomatic bone metastases in the thorax, FAST‐02, has started patient accrual (https://clinicaltrials.gov/study/NCT05524064). More clinical trials are foreseen growing towards a mature clinical treatment modality. This means that clinics need to prepare their clinical workflow, which also includes patient‐specific pre‐treatment verification. Pre‐treatment verification in conventional radiotherapy focusses on dose and geometry. However, within FLASH radiotherapy also dose rate is an important parameter and therefore fast time‐resolved detectors are required.^6^

This study has a focus on pencil‐beam scanning FLASH proton therapy for which the spatiotemporal structure of beam delivery is complex. Individual spots are delivered in the order of milliseconds^7^ while scanning the target, one spot after the other. A complete treatment field is typically delivered in a fraction of a second.^6^ Due to this complexity the definition of dose rate is not straight forward. Several definitions have been proposed, currently the PBS dose rate definition^8^ seems to be the most used. It is defined as the ratio between the delta dose (ΔD) and delta time (Δt), depending on a predefined threshold, for a given position.

Most clinics use a 2D array of ionization chambers for patient‐specific QA measurements. These detectors do have the possibility to perform time resolved measurements; however, the spatial resolution (∼7 mm) and temporal resolution (20–500 ms per frame) is low, also these detectors saturate at high dose rates and/or high dose values. Spot delivery times are in the order of milliseconds, meaning that ideally the temporal resolution of the detector is equal or smaller. Furthermore, the PBS dose rate distributions can be very inhomogeneous ranging between tens up to hundreds of Gy/s over a distance of a few millimeters, mostly depending on spot pattern and nozzle current. Therefore, is it important to achieve a submillimeter spatial resolution to avoid an averaging effect that occurs when a relatively large sensitive measurement area is used in an inhomogeneous distribution.

There are new detectors being developed, capable of measuring UHDR proton beams; for instance strip detectors, for example, CROSS_mini_ SICA detector (Liverage Biomedical Inc., Hsinchu, Taiwan) and FlashDose (DE.TEC.TOR, Turin, Italy), and scintillation based detectors.^9^, ^10^ From the previously mentioned detectors only the strip detectors are commercially available at this point in time. Despite the high temporal resolutions of these detectors, 50–100 µs per frame, the spatial resolution is limited with a 2–3 mm spacing between the strips. Another disadvantage is that only the spot position can be determined, by pre‐defining a spot shape the 2D accumulation over time can be simulated. An alternative detector to assess the time structure of beam delivery could be Gafchromic film which has an unmatched spatial resolution, and, with a reaction time in the order or micro seconds.^11^ The mechanism of Gafchromic films, consisting of monomers in the active layer, is based on creating radiation induced polymers causing the film to turn blue (coloration). An increase in delivered dose to the film results in an increase of coloration, which continuous to develop slowly even after the beam delivery has stopped. This phenomenon is known as the post‐irradiation coloration effect.^12^ It is important to take this effect into account, as it can lead to an overestimation of the delivery time. Additionally, it is also known that the film response is LET dependent. However, in case of transmission beam FLASH this aspect can be ignored due to a constant LET at the plateau region.

Our main aim is to develop clinically feasible patient‐specific pre‐treatment verification on the time structure for FLASH proton therapy. To this end, we assess the feasibility of measuring the time structure of beam delivery, Δt, with Gafchromic film, imbedded in a setup integrated with a high‐speed camera. In the following, we first explain the concept of time resolved film dosimetry (TRFD). Second, the correct determination of Δt is verified by comparing the film measurement with a reference detector. Third, we exclude that revisiting spots have a negative impact on Δt. Finally, we determine the level of agreement between measurement and simulation for a set of QA plans and three clinically relevant treatment plans, the latter for both PBS dose rate optimized and non‐optimized spot patterns.

## 2 MATERIALS AND METHODS

### 2.1 Beam delivery

VARIAN's Probeam 4.0 gantry platform (VARIAN, Palo Alto, USA) was used to deliver 250MeV UHDR scanning proton beam in combination with Racehorse 2.0 (VARIAN, Palo Alto, USA). Nozzle currents up to 215nA were available.

Beam delivery on the gantry requires a so‐called spot list. It is a plain text file in which per line all information per spot is defined, being the x‐position, y‐position, and MU. The chronological order of the spot list dictates the order at which spots are delivered. The gantries monitor chamber saturates at a nozzle current of ≥20 nA, therefore the FlashQ (DE.TEC.TOR, Turin, Italy), an ionization chamber‐based detector, was positioned in between the beam and measurement setup for all measurements to monitor the delivered integrated dose in arbitrary units over time at 1 kHz.

### 2.2 Concept of time resolved film dosimetry

#### 2.2.1 Detector design

**FIGURE 1:**
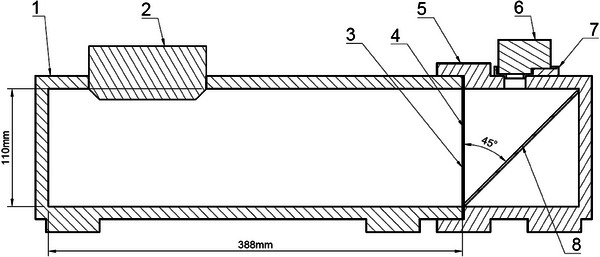
Design of detector setup to support time resolved dosimetry. To avoid ambient light an enclosed environment (1, 5) was created having its own light source (2). The light passes through a 1 mm polystyrene plate (3) to assure a diffuse light exposure to the film (4). A camera (6) in a holder (7) captures the coloration of the film via a mirror (8). The film center is aligned to the gantries isocenter, beam entered from the right side passing through the mirror before reaching the film.

To facilitate TRFD, a Hero 10 camera (GoPro, San Mateo, USA) camera was used to capture the radiation induced coloration process of Gafchromic film. A 45° tilted mirror with respect to the central beam axis was used to avoid radiation damage to the camera (Figure 1). To control the light conditions an in‐house build enclosed multiplex wooden box was made which contained a direct voltage light source to avoid light intensity oscillations. A film was positioned in between the mirror and the light source. To maximize diffuse light onto the film a 1 mm white polyethylene (PE) sheet was positioned next to the film, and, the inside of the box on the same side was spray painted with a structured chromic color spray‐paint. The camera's effective measurement area of the film is 9 × 9 cm^2^ with a resolution of 640 × 640 pixels (∼0.14 mm per pixel), meaning that only a selection of the camera's beams eye view is used.

EBT XD films with lot number 01182201 (Ashland Inc, Bridgewater, NJ, USA), dimensions 8 × 10 inch^2^, were used for all experiments. From each film two cuts of 135 × 127 mm^2^ were taken to fit into the detector. A RUFUS 1500 MA (Brennenstuhl, Tübingen, Germany) was used as light source (1500 lm), despite the option that it can run at the internal battery, the light source was used connected to a power adapter at all times. To assure a stable illumination a 3.5 h warmup time of the light source was respected.

The following camera settings were selected; resolution of 1800x, 240 fps, lens set to linear to correct for barrel distortion, HyperSmooth (stabilizing footage for motion) was turned off, bit rate high, shutter 1/240, iso min and max were set to 100, white balance native, sharpness low, color flat. All settings were chosen to have the least color corrections and a maximum temporal resolution. The duration of each recording continued at least 60 s after start beam (beam delivery time is in the order of 1 s) to assure that the local variations in the post‐irradiation coloration effect of the film were minimized (stabilization time), due to spot per spot delivery and thereby the varying starting point of the film coloration in time. The latter is important to assure a stable relative dose determination per pixel value. All recorded footage was stored as a mp4‐file, each frame consisting of a 24‐bit color image from which only the red color channel (8 bit) was used for further analysis.

##### Light source stabilization time

A constant illumination is paramount in film dosimetry, for that reason the luminance (cd/m^2^) was measured for 6 h with time intervals ranging from 0.5 to 1.0 h at the center of the PE plate. Measurements were performed with the Accu‐Gold (Radcal, CA, USA) system used in combination with the light sensor including the luminance adapter.

#### 2.2.2 Post processing step 1/3: Transform reading into relative dose

This section and the sections that follow will discuss all post‐processing steps. Section 2.2.2 explains how the relative dose is obtained from the time resolved film measurement. How to correct the obtained dose over time measurement for the post‐irradiation coloration effect is presented in Section 2.2.3. The last post‐processing step, Section 2.2.4, is to calculate the PBS dose rate.

**FIGURE 2:**
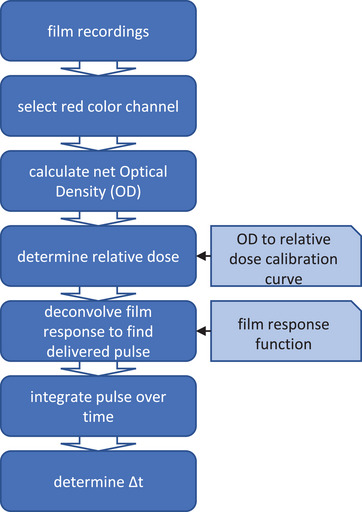
Flow chart displaying all post processing steps to get from the recorded radiation induced coloration of the film up to the Δt value per pixel position. Two calibration curves are required during the process; the OD to dose calibration curve and the film response function.

Similar to conventional film dosimetry several post processing steps are required to transform the pixel value (PV) of the red color channel into dose (Figure 2). First the optical density (OD) was determined using the following equation:

(1) $$OD=\;\log\frac{2^{b}}{\mathrm{PV}+1}$$

Variable b depends on the bit size of the color channel, which is 8 in our situation. Since the camera recordings start before beam delivery, the net OD (OD_net_) was calculated for each frame by subtracting the OD from the first frame. The relation between OD and dose, OD to dose calibration curve, was determined using a polynomial fit based on the following equation^13^:

(2) $$\;D_{fit}=a+b•OD_{net}+c\;•O{D_{net}}^{m}$$

Section “Create OD to dose calibration curve” explains which dose steps were used and how the corresponding OD values were obtained. After applying D_fit_ to all calculated OD_net_ values the end result is a time resolved accumulation of 2D relative dose, still affected by the post‐irradiation coloration effect.

##### Create OD to dose calibration curve

Nine films were used, each receiving one of the following dose levels; 0.0, 0.5, 1.0, 2.0, 5.0, 10.0, 20.0, 30.0, and 50 Gy. For dose delivery a 30 × 30 mm^2^ spot pattern was used, 5 mm spot spacing and a uniform MU weight per spot position. For each dose level the MU weight per spot was changed accordingly. Each film was placed at 3 cm depth in a 5 cm thick RW3 slab phantom (PTW, Freiburg, Germany), the phantom surface aligned to the isocenter. To correct for minor variations in delivered dose an Advanced Markus ionization chamber (PTW, Freiburg, Germany) was positioned directly behind the film on the central beam axis. An UNIDOS E electrometer (PTW, Freiburg, Germany) was used to measure the collected charge using a voltage of 400V, corrections for temperature and pressure were applied.

After 24 h post processing time, all films were positioned in the time resolved measurement setup, recoding each film for ∼20 s. Per film 10 s of footage (2400 frames) was selected and averaged at a time point halfway the recording. A region of interest (ROI) of approximately 1 × 1 cm^2^ in the center of the irradiated field was taken to determine the average PV for the red color channel, the OD was determined using Equation (1). With both the OD_net_ and dose values known it was possible to fit the OD_net_ to dose calibration curve using Equation (2) (D_fit_).

#### 2.2.3 Post processing step 2/3: Undo post‐irradiation coloration effect

To undo the post‐irradiation coloration process, the assumption is made that each deposit of dose over time results in a similar film response over time. By knowing the film response for an infinite small pulse, that is, the film response function, it becomes possible to correct for the post‐irradiation coloration effect for each PV_x,y_. This is performed by deconvolving the film response by the film response function resulting in the delivered pulse. The film response and film response function are described in Equations (3) and (4), respectively.

(3) $$\begin{array}{ccc} & & film\;response_{x,y}\;\;\left(t\right)=pulse_{x,y}\;\;\left(t\right)\\ & & \;⊗\;film\;response\;function\;\left(t\right) \end{array}$$

(4) $$film\;response\;function\;\left(t\right)=offset+c\;\cdot \;log_{10}\left(t+a\right)$$

(5) $$relative\;dose\;accumulation\;\left(t\right)=\int_{0}^{t}pulse_{x,y}\;\left(t^{\prime }\right)\;dt^{\prime }\;$$

Finally, the relative dose accumulation over time is given by the integral over the pulse, Equation (5).

All data were handled by a Python script, convolve and deconvolve steps were performed using *convolve* and *deconvolve* from the *Scipy.Signal* library. For the integral over time the *numpy* function *cumsum* (cumulative sum) was utilized.

##### Obtain film response function

A single spot with a maximum dose of 20 Gy was delivered with a nozzle current of 215 nA using time resolved dosimetry. The dose accumulation over time was determined for all PV within the 50%‐isodose line (ICRU treatment field). All PV were averaged per time step, resulting in a 1D data set representing the relative dose accumulation over time. An automated fitting function created in python was used to fit the film response for a known block pulse, given by the nozzle current in nA over time, in line with Equations (3) and (4). The nozzle current and pulse duration were obtained from the reference detector (FlashQ) measurements. Finally, a set of fitting parameters was obtained for the film response function.

#### 2.2.4 Post processing step 3/3: Determine PBS dose rate

The PBS dose rate definition of Folkerts et al.^8^ was selected to determine the dose rate distribution. The PBS dose rate is defined as follows: for each position (pixel or voxel) dose will accumulate over time from zero (D_min_) to the dose maximum (D_max_). Next, a dose threshold (d) is defined, for example, absolute dose of 0.1 Gy or a certain percentage of D_max_, by the user to determine the starting point, D_min_ plus d, and the stopping point, D_max_ minus d, on the dose accumulation curve. The difference between the points in dose and time provides, respectively, delta dose (ΔD) in Gy and delta time (Δt) in s, PBS dose rate in Gy/s is then given by the ratio of ΔD and Δt. TRFD is used to determine Δt, while conventional film dosimetry could be used to determine ΔD.

##### Determine delta t

To find Δt per PV, the dose accumulation over time was normalized to 100% for the last averaged 240 frames (equal to 1 s) representing the final and maximum dose per PV. Next, the two thresholds were set at 10% and 90% (d = 10%) of the maximum dose of that same PV to find the times of intersect, t1 at 10% and t2 at 90%, using linear interpolation. The absolute difference between the two points gives the Δt value.

##### Determine delta dose using conventional film dosimetry

At this point only the relative PBS dose rate, based on relative dose accumulations over time, could be determined since the TRFD setup was not calibrated for absolute dose. In the current form it is still the idea to rely on conventional film dosimetry to calibrate the TRFD measurements to absolute dose. However, this step is left out of scope for this study.

### 2.3 Simulation of dose delivery over time

For the final validation tests the measured 2D Δt distributions were compared to simulated 2D Δt distributions. In absence of a FLASH treatment planning software a 2D dose over time simulation python script was made. For commissioning the following parameters were required:

- Spot shape: A normally distributed spot shape was assumed
- Spot size: The spot size within the time resolved film measurement setup was determined by delivering a single spot of 6 Gy on Gafchromic film for nozzle currents of 5, 25, 120, and 215 nA. All films were post processed according to the conventional film as highlighted in Section 2.3.1, resulting in four 2D dose distributions. A 2D gaussian distribution was fitted onto the 2D dose distributions to determine the sigma of each spot. The average spot sigma was used as input for simulation. A value of 3.8 mm was used for all further simulations.
- Dose per MU: Since the monitor chamber of the VARIAN Probeam system tends to saturate a nozzle current above 20 nA a linear fit was made to correlate dose per MU for a given nozzle current. To do so a series of 3 × 3 cm^2^ spot patterns with various combinations of dose (12, 15, and 20 Gy) and nozzle current (25, 120, and 215 nA) were delivered onto an Advanced Markus within the TRFD setup at the position of the film. The measurement series were repeated four times to take into account fluctuations in nozzle current. With these measurements only the field dose is known. To go from field dose to the maximum dose of a single spot, the spot shape and spot size were used to simulate the relative dose distribution for the applied spot pattern and for a single spot of that same spot pattern. The ratio between the two was used to determine the dose for the single spot. The last step was dividing the dose for a single spot by the number of MU for that same spot.
- Time per MU: Due to the saturation effect of the monitor chamber the time per MU is constant above nozzle currents of 20 nA. During the dose per MU measurement, all fields passed through the FlashQ from which the delivery time per field was obtained. Finally, the average time per MU was applied.

Dose accumulation over time could then be simulated by entering a spot list as discussed in Section 2.1. The spatial and temporal resolution were set at 0.1 mm and 10 kHz, respectively. Finally, 2D Δt distributions were calculated using the same method for PBS dose rate calculation as defined in Section 2.2.4.

#### 2.3.1 Determine spot shape

In Section 2.3 conventional film dosimetry was required to determine the spot shape. To do so an in‐house script was made to determine the dose distribution of an irradiated film. Each film (48‐bit, 150 dpi, no color corrections) was scanned 24 h post irradiation using an Expression 12000XL flatbed scanner (Seiko Epson Corporation, Nagano, Japan) in transmission mode in combination with the Epson Scan 2 software (Seiko Epson Corporation, Nagano, Japan). To obtain the required OD to dose calibration curve the same OD to dose calibration films as in Section “Create OD to dose calibration curve” were used. Each film was scanned at five equidistant lateral scanning positions to not only take into account the OD to dose relationship but also the lateral scan effect. A 2D polynomial fit was used, third order to find the relation between OD and dose and a second order for the lateral scan effect.

### 2.4 Validation

#### 2.4.1 Correct determination of delta t

Nine single spots with various combinations of dose (5, 10, and 20 Gy) and dose rate (25, 120, 215 nA) were delivered and measured using TRFD to determine the Δt distribution. Only those PV with a final dose above the 50%‐isodose line were taken into account. The average Δt values were compared to the Δt obtained by the strip detector.

#### 2.4.2 Evaluate potential impact of revisiting spots

Three spot patterns were created each consisting of only 3, in series over the y‐direction, target positions for spot delivery 15 mm apart (y‐positions: +15.0, 0.0, and −15.0 mm), where each target position finally received a dose of 10 Gy. The spot list itself consisted of 3, 6, and 9 spots delivered in a snake pattern starting at the +15 mm y‐position, without making a lateral shift in the x direction. This approach resulted in center target position receiving the 10 Gy dose in either once (three spots spot pattern), twice (six spots spot pattern), or thrice (nine spots spot pattern). Per spot pattern, the MU weight per spot was equally distributed. This was repeated for nozzle currents 25, 120, and 215 nA resulting in nine measurements in total.

For further analysis, the average in‐field (defined by the 50%‐isodose line) PV was used for creating the relative dose‐over‐time curve. All nine curves were scaled to 1 at the 60 s time point past start beam and compared to reference relative dose lines. These reference lines are positioned at the expected relative dose levels corresponding to a pause between two consecutive spot deliveries. A 1/3 and 2/3 reference lines were created for the deliveries in thrice, a 1/2 reference line was used for the delivery in twice, no reference line was added for the delivery performed once. A qualitative comparison between measurement and reference lines was performed by visually comparing the accumulated dose per spot to the expected accumulated dose.

#### 2.4.3 Measurement versus simulation for QA plans and clinical plans

##### QA plans

Nine QA plans consisting of a 3 × 3 cm^2^ spot pattern, spot spacing 5 mm with equally MU weighted spots, were created with different combinations of dose (12, 15, and 20 Gy) and nozzle current (25, 120, and 215 nA). Each plan was measured using the TRFD measurement setup and the 2D Δt distributions were obtained for the in‐field PV. For each field, also the simulated 2D Δt distributions were calculated and linearly scaled in time by using the measured beam delivery time obtained by the FlashQ. Both Δt distributions were compared using a local gamma evaluation after matching the corresponding dose distributions first (not the Δt distribution), gamma criteria being 10% (in Δt) and 2 mm. For each plan the gamma passing rate is reported.

##### Optimized patient specific plan

José Santo et al. have shown in 2019 that by changing the order of spot delivery, where snake or z pattern is standard in conventional PBS proton therapy, it is possible to increase the PBS dose rate referred to as spot pattern optimization.^14^ It also included a dose‐optimized treatment plan, based on a 3D CT scan, consisting of three 244 MeV beams. The spot delivery order was optimized to maximize the PBS dose rate. For our study we used both the spot pattern optimized and non‐optimized plans, six beams in total, after changing the beam energy to 250 MeV and normalizing the dose on the central beam axis to 18 Gy. As for the QA plans, the maximum dimensions of the spot pattern was 3 × 3 cm^2^ in combination with a 5 mm spot spacing. Each beam was considered as a clinically relevant treatment plan and was evaluated similarly as the QA plans.

## 3 Results

### 3.1 Light source stabilization time

The luminance decreased gradually from 486 to 435 cd/m^2^ over a time period of 3.5 h after which it stayed constant.

### 3.2 OD to dose calibration curve for time resolved film dosimetry

**FIGURE 3:**
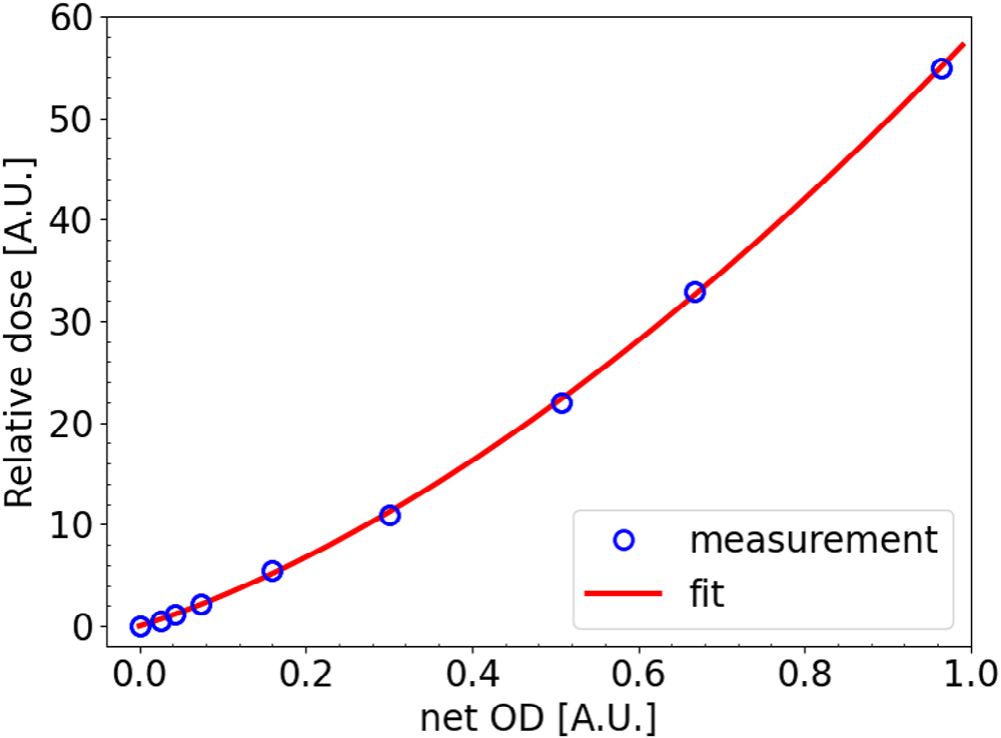
Obtained fit to correlate relative dose to OD_net_.

Figure 3 shows the measurement results and obtained fit. Obtained fitting parameters were −7.48*10^−3^, 24.5, 33.5, and 1.80 for a, b, c, and m, respectively.

### 3.3 Derived film response function

**FIGURE 4:**
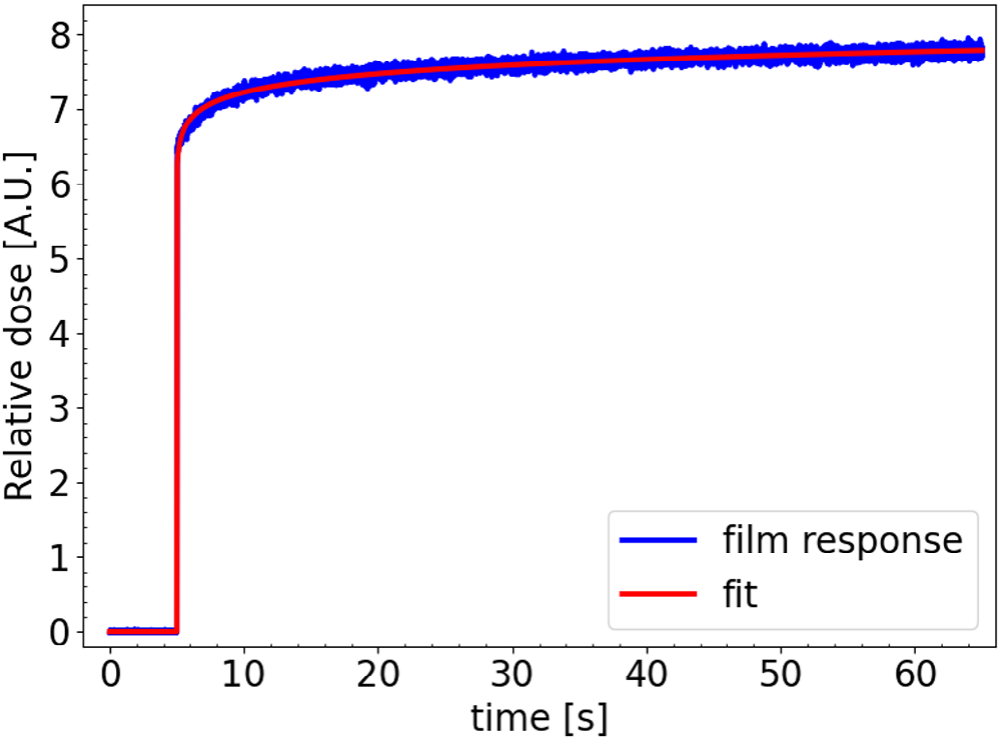
The average in‐field film response for a single spot, maximum dose 20 Gy and nozzle current 215 nA. Also, the obtained fit is given.

**FIGURE 5:**
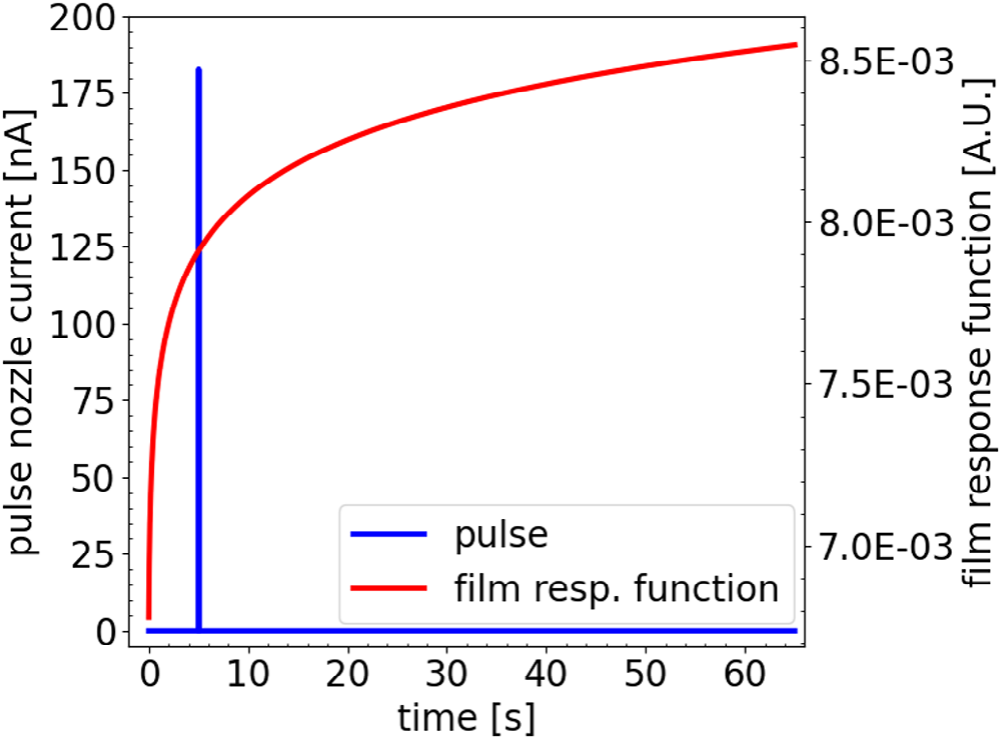
Obtained film response function and applied pulse.

**FIGURE 6:**
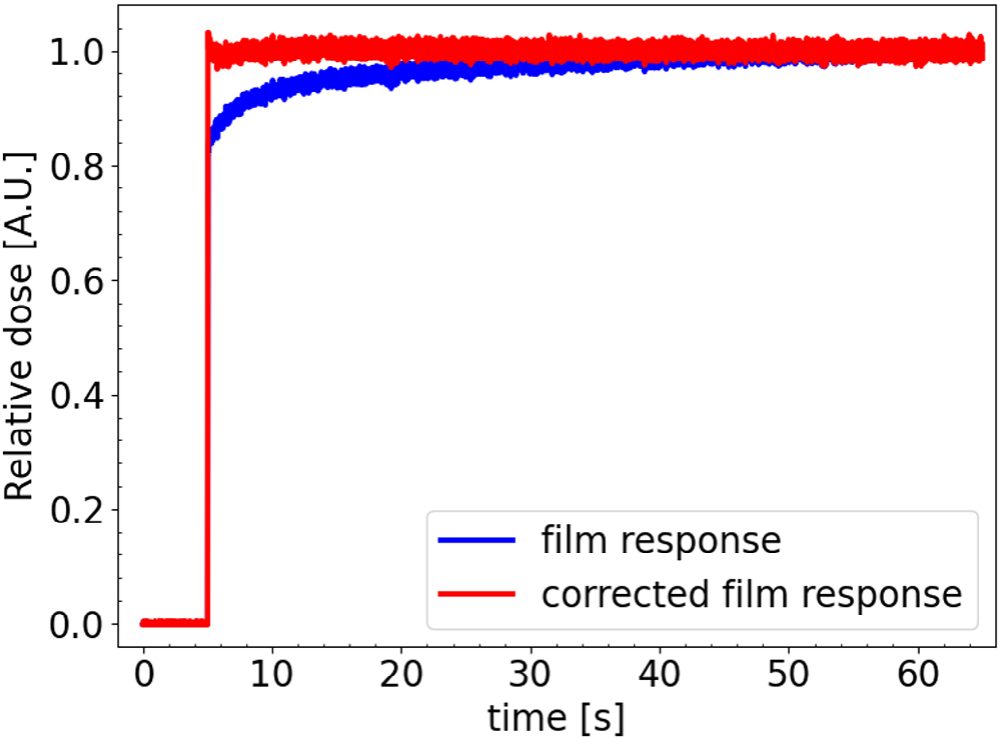
Comparison between film response as presented in Figure 4 and the corrected (deconvolved) film response using the film response function as displayed in Figure 5.

Figure 4 shows the average in‐field film response of a single spot of 20 Gy delivered at a nozzle current of 215 nA. The obtained fit, derived from Equation (3), is also included in the figure. Figure 5 shows the corresponding pulse and film response function. Although a 215 nA nozzle current was requested, the FlashQ reference detector indicated a lower nozzle current being 183 nA. The obtained fitting parameters of Equation (4) are 7.5*10^−3^, 5.4*10^−2^, and 5.7*10^−4^ for, respectively, offset, early response gradient (a), and late response gradient (c). Figure 6 demonstrates the impact of correcting for the post‐irradiation coloration. It contains an original film response for a single pulse, the same as presented in Figure 4. In addition, the corrected film response is presented using the film response function as presented in Figure 5. To facilitated a fair comparison between both curves, original and corrected film response, were scaled both curves to 1 at the 60 s time point past start beam delivery.

### 3.4 Spot size for dose simulation

The average spot sigma was determined at 3.73 and 3.79 mm for a nozzle current of 5 and 25 nA, respectively, a value of 3.81 mm was found for both 120 and 215 nA. The overall spot sigma was averaged and determined at 3.8 mm, used as input for dose simulations.

### 3.5 Correct determination of Δt

**FIGURE 7:**
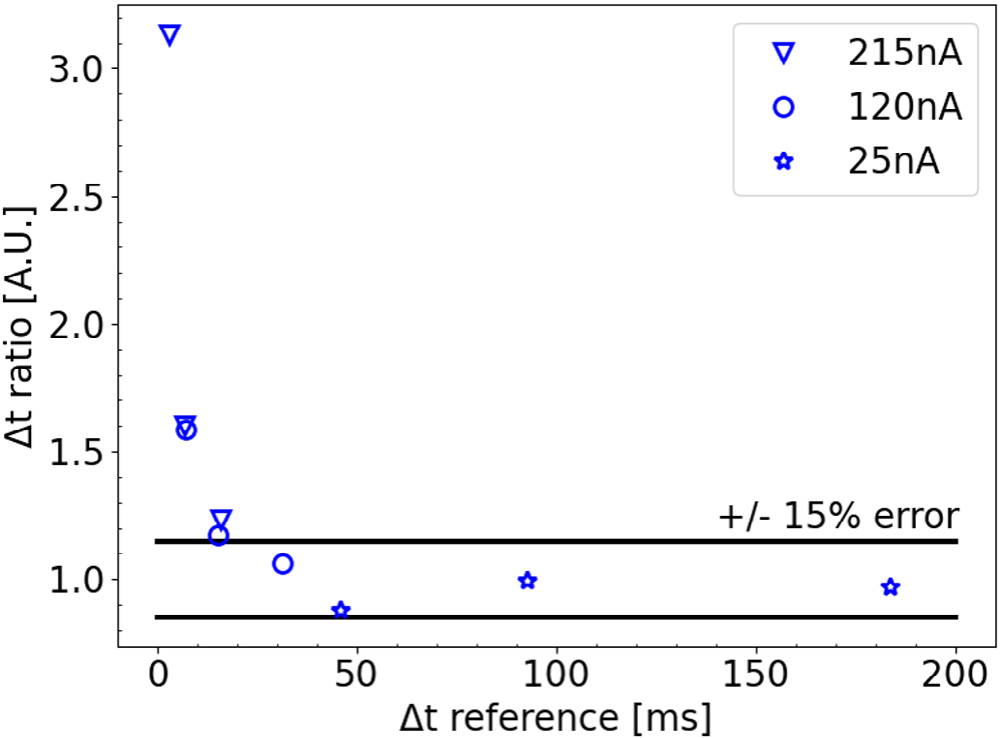
Comparison between film and reference detector (FlashQ), for the determination of Δt for various single spots. The y‐axis shows the ratio (film Δt /reference Δt) against the reference Δt values on the x‐axis. An error limit of ±15% was set corresponding to the horizontal threshold lines at Δt ratio equal to 0.85 and 1.15.

The Δt values for nine spots, various nozzle currents and dose levels, were measured using time resolved film and compared to the reference detector (FlashQ). Figure 7 shows the ratio between both with respect to the reference Δt value. The results show that TRFD is capable to determine Δt accurately; however, for Δt values <20 ms the error with respect to the reference becomes more than 15%.

### 3.6 Evaluation of the potential impact of revisiting spots

**FIGURE 8:**
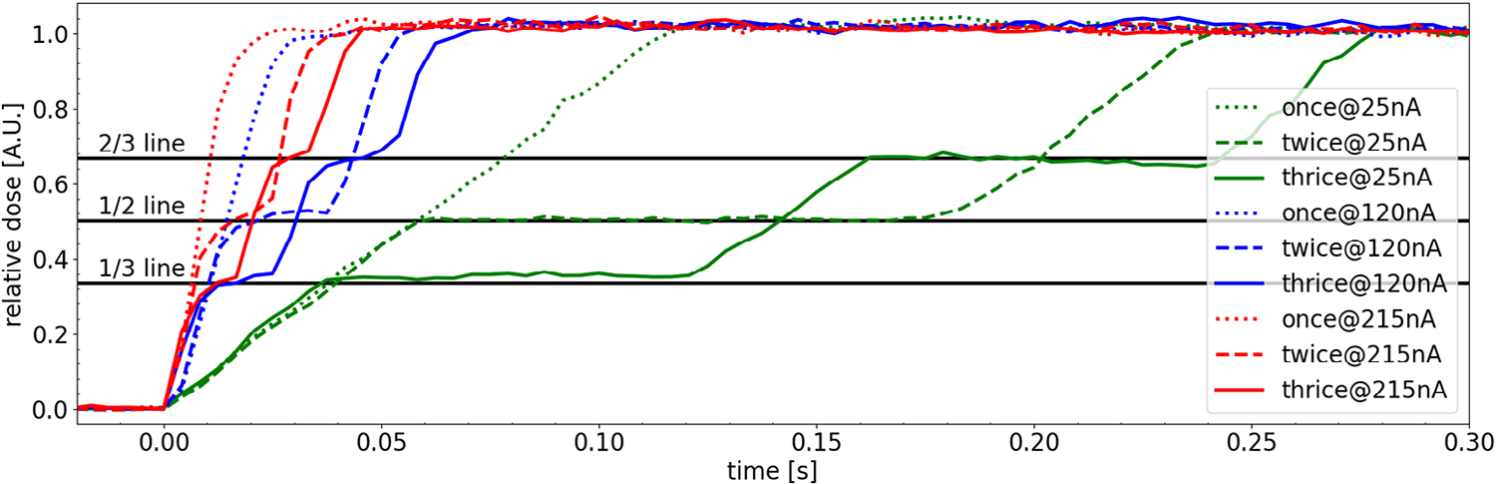
Nine spots with a maximum dose of 10Gy were delivered in one (once), two (twice) or three (thrice) equally divided dose steps with a pause in between. The dose level at each pause corresponds to the expected dose levels (1/3, 1/2, and 2/3 line). The measured dose rate remained unaffected (see, e.g., the similar slopes of the dashed lines).

Figure 8 shows the relative dose accumulation over time for spots being delivered in one (once) or two (twice), or three (thrice) equivalent dose steps. For the latter, two pauses in the delivery were included at the 1/3 and 2/3 line. For the delivery in twice, the pause should be at the 1/2 line. It can be seen that for this validation test the pauses are found at the expected relative dose point, independent of the applied nozzle current. Another observation is that also the dose accumulation gradient seems to be similar for each spot that was delivered at a specific nozzle current. These findings show that time‐resolved measurements are not impacted by revisiting previous irradiated spots.

### 3.7 Measurement versus simulation for both QA and clinical treatment plans

Table 1 shows all results regarding the comparison between film measurements and simulations for both QA and clinical treatment plans. The gamma index for the local gamma evaluation (у_local_) shows good agreement between measurements and simulations, with a gamma index ≥ 0.90 for all evaluated treatment plans. The same is true for the higher Δt values within the 2D Δt distributions looking to the 95th percentile (p95). On the other hand, there seems to be a dependency for the lower Δt values, more pronounced for the 5th percentile (p05) compared to the 50th percentile (p50), showing an underestimation for relative longer overall delivery times. With an exception for plan A1, shortest overall delivery time, showing an overestimation.

**TABLE 1:** Overview of evaluated treatment plans.

		QA treatment plans									Clinical treatment plans					
Plan	Name	A1	A2	A3	A4	A5	A6	A7	A8	A9	B1_opt_	B1_ori_	B2_opt_	B2_ori_	B3_opt_	B3_ori_
	D [Gy]	12	15	20	12	15	20	12	15	20	18	18	18	18	18	18
	I_n_ [nA]	215	215	215	120	120	120	25	25	25	40	40	40	40	40	40
у_local_	^2mm^/_10%_	0,90	0,98	0,98	0,96	0,99	0,98	0,97	0,98	0,93	0,97	0,95	0,99	0,98	0,98	0,97
Δt_m_ [s]	Mean	0,043	0,076	0,410	0,054	0,097	0,508	0,067	0,116	0,622	0,302	0,312	0,334	0,313	0,317	0,312
	p5	0,019	0,029	0,141	0,022	0,036	0,173	0,028	0,044	0,219	0,114	0,162	0,117	0,179	0,114	0,168
	p50	0,049	0,089	0,478	0,061	0,113	0,586	0,075	0,127	0,675	0,217	0,294	0,198	0,292	0,186	0,290
	p95	0,056	0,099	0,555	0,069	0,124	0,687	0,089	0,160	0,890	1,139	0,468	1,203	0,455	1,113	0,461
Δt_s_ [s]	Mean	0,043	0,080	0,457	0,055	0,101	0,571	0,073	0,134	0,763	0,349	0,368	0,367	0,358	0,351	0,362
	p5	0,015	0,029	0,166	0,020	0,038	0,208	0,027	0,049	0,278	0,126	0,193	0,125	0,196	0,125	0,189
	p50	0,050	0,093	0,529	0,063	0,117	0,661	0,085	0,155	0,882	0,241	0,356	0,217	0,361	0,202	0,339
	p95	0,052	0,096	0,547	0,065	0,121	0,684	0,088	0,161	0,914	1,155	0,483	1,201	0,466	1,113	0,471
Ratio	Mean	1,00	0,95	0,90	0,98	0,95	0,89	0,91	0,86	0,82	0,86	0,85	0,91	0,88	0,90	0,86
	p5	1,26	1,01	0,85	1,12	0,97	0,83	1,06	0,90	0,79	0,90	0,84	0,94	0,91	0,91	0,89
	P50	0,97	0,96	0,90	0,97	0,97	0,89	0,89	0,82	0,77	0,90	0,83	0,91	0,81	0,92	0,85
	p95	1,07	1,03	1,01	1,05	1,03	1,00	1,02	1,00	0,97	0,99	0,97	1,00	0,98	1,00	0,98

*Note*: For each plan the following data are presented: generic plan parameters which includes plan name, normalized dose to central axis (D) and the applied nozzle current (I_n_). For comparison the outcome of the local gamma evaluation (у_local_) is added. Additionally, also several Δt distribution statistics (mean and 5th, 50th, 95th percentile) are presented for both film measurement (Δt_m_) and simulation (Δt_s_), including the ratio of both (Δt_m_/Δt_s_).

**FIGURE 9:**
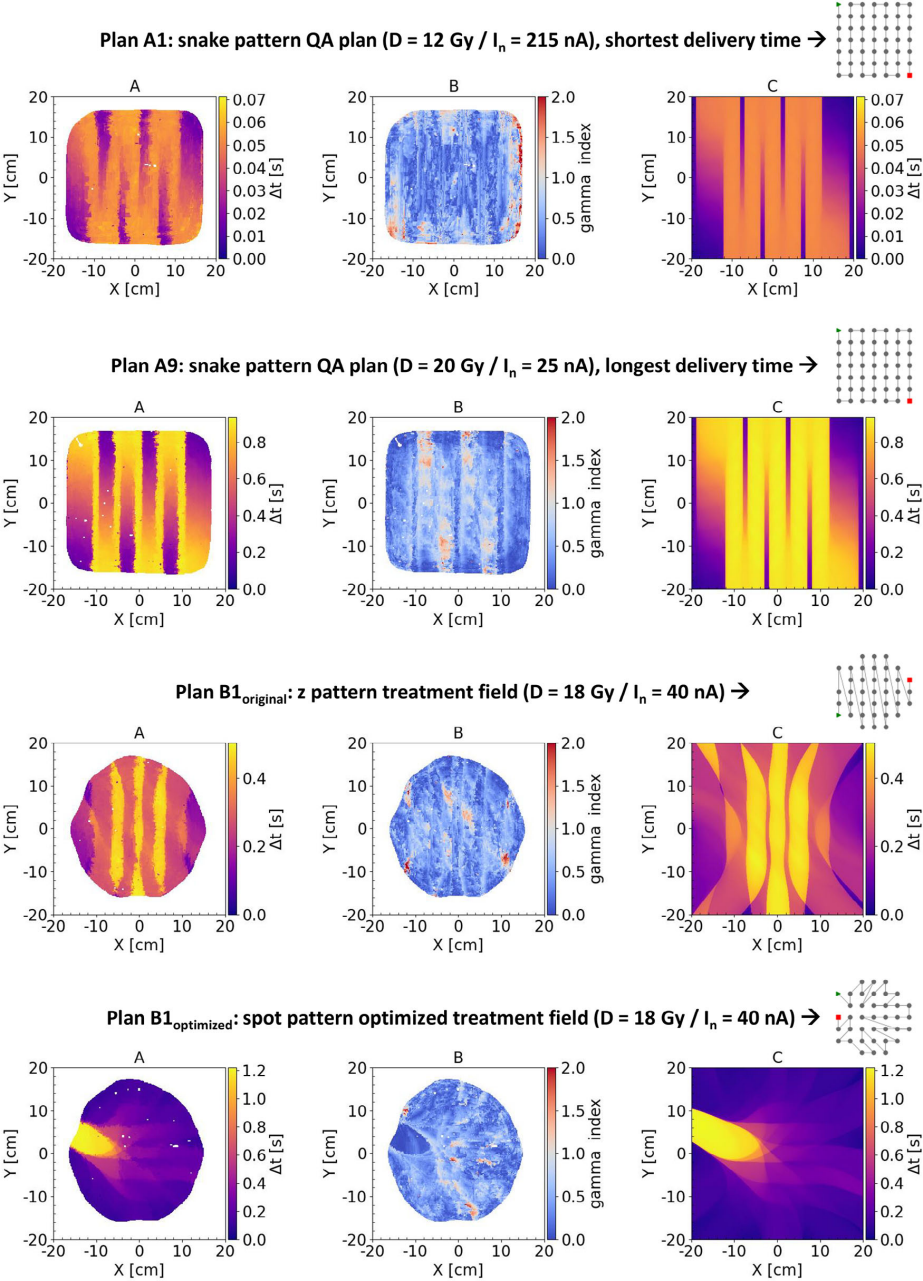
Four examples, two QA plans (A1, A9) and two clinical plans (B3_ori_, B3_opt_), of the local gamma evaluation (B), gamma criteria 2mm/10%, comparing the measured (A) to the simulated (C) 2D Δt distribution. Only in‐field pixel values were evaluated.

To obtain more insight in what kind of differences are observed between measurements and simulations, the 2D local gamma evaluations, including the measured and simulated Δt distributions are presented in Figure 9 for QA plans A1 and A9, and for clinical plans B1_ori_ and B1_opt_. In general, less agreement, high gamma values, is found for low Δt regions and steep gradients from low to high Δt values and vice versa. The latter is mostly visible for the plans based on snake or z pattern delivery (A1, A9, and B1_ori_). Especially for plan A9 it is visible that the vertical bands of high dose rates are smaller for the film measurement compared to the simulated Δt distribution, resulting in a lower p5 and p50 value.

## 4 Discussion

In this study, we have experimentally measured the dose delivery time structure down to a millisecond timescale for a gantry based UHDR scanning proton beam. It has been shown that it is feasible to perform patient‐specific pre‐treatment verification using time resolved film dosimetry (TRFD).

The current TRFD system performs near‐surface measurements and has only been tested for transmission beams. However, it is expected to be valid for Bragg Peak FLASH as well, based on, for example, 2D or 3D ridge filters.^15^ For the latter it is an important prerequisite that the Spread Out Bragg Peak (SOBP) lies beyond the near‐surface measurement depth to avoid LET dependency of Gafchromic film. In depth measurements might be feasible provided the following two points are addressed: adaptation of the detector design to facilitate measurements at a specific depth and a method to correct for LET dependency. By positioning transparent slabs, for example, PMMA, in front of the PE plate, it is possible to vary the measurement depth. For this, it is important that the beam enters from the opposite side of the detector compared to what was done in this study, allowing the beam to pass though the slabs first before reaching the film. Regarding LET dependency, the study by Anderson et al. demonstrated a linear relationship between film response and LET for EBT3 in conventional film dosimetry.^16^ In addition, they proposed a method to correct for LET dependency. This method could also be applied for EBT XD in TRFD, however, requires further research.

The current detector design uses an easily accessible, non‐professional, camera with a temporal resolution of 240 fps (∼4 ms/frame). Knowing that the minimum spot duration for the proton delivery system is 3 ms, the current camera seems suboptimal. This is reflected in Figure 7, which shows an overestimation of ∆t for ∆t values < 20 ms. Despite this limitation, good results are obtained in the gamma evaluation for the clinical and QA plans (see Table 1 and Figure 9). The reason for the positive results is the following: treatment fields consist of multiple spots that partially overlap to create a homogeneous dose distribution. As a consequence, the dose for a given position is composed of contributions from multiple spots, making the total beam time several multiples longer than of a single spot. Another factor contributing to the total beam time being longer than that of a single spot is the so‐called dead time, during which spots are delivered elsewhere in the field before returning to the pixel in question to deliver the remainder of the dose. From our results, it can be concluded that the current camera with a frame rate of 240 fps is sufficient for QA purposes of clinical plans. However, if necessary, the temporal resolution can be easily increased by replacing the current camera with one with a higher frame rate.

In addition, a constant illumination of the film is paramount in film dosimetry since it has a direct impact on the measured dose, meaning that the light intensity of the light should be constant. However, the applied light source required a warming‐up time of ∼3.5 h before the light intensity stabilized. It seemed to be dependent on the temperature of the light source itself, which slowly increased over time. This temperature increase was only monitored by touching it by hand, no temperature measurements were performed. The setup did not include active cooling, which most likely will decrease the stabilization time. Other options would be to use an alternative light source for which light intensity is less affected by temperature or has an active feedback loop to assure light intensity stability.

A limitation in the concept of TRFD is that the time resolved film measurement outcomes heavily depend on the retrieved film response function. From a theoretical point of view the applied pulse to determine the film response function should be as short as possible. However, since the camera introduces noise, the signal to noise ratio (SNR) is rather low resulting in a less accurate fit of the film response function. By increasing the pulse duration at the maximum nozzle current, the SNR was sufficiently high to find an accurate fit of the film response function. These pulse settings may vary depending on the camera type used, for example, a more high‐end camera may require a shorter and lower intensity pulse to reach a similar SNR.

A comparison between this study and the study of ref. ^17^, which has shown the feasibility of log file‐based patient‐specific pre‐treatment verification. The log files were derived from strip detector measurements, as a surrogate gantry monitor chamber, and used to reconstruct dose and PBS dose rate in a 3D‐CT scan. In their method, based on measurement with high temporal resolution (50 µs per frame), but low spatial resolution (2 mm), still assumptions on the spot shape and interpolation to achieve a higher spatial resolution are required. TRFD measurements are on the other hand a more direct measurement method, meaning no interpolation or assumption of the spot shape has to be made. A joint finding is that the gamma evaluation is a proper method to compare two PBS dose rate distributions. To our opinion a local gamma evaluation is needed due to the large inhomogeneities present in the PBS dose rate distribution where the minimum PBS dose rates are most important to reach the desired FLASH effect. In addition, the spatial gamma criteria should be taken at a low value, for example, 1–2 mm, to be sensitive enough to evaluate the small peaks and valleys in the PBS dose rate distributions as presented in Figure 9.

An important discussion point is the suggested dose threshold (d) within the PBS dose rate definition by Folkerts et al., which has a large impact on the calculated PBS dose rate. Their study, based on time resolved dose simulations, states to use a relatively low absolute dose value, for example, 0.1 Gy. However, there are three reasons why choosing d correctly is important. First, the dose accumulation for an in‐field position could in general be described by a sigmoid curve, having a low increase in dose when the scanning beam is far away (low gradient) and fast increase when the scanning beam is nearby (high gradient). Minor variations for a relative low d value will cause large fluctuations in the time component of the PBS dose rate, while relative high d values, in the high dose increase region, will cause large fluctuations in the dose component. Using a d value in the transition region going from a low to high gradient, provides a more stable point regarding both time and dose and thereby the PBS dose rate. For our study a d value of 10% seemed to be working reasonable; however, this also depends on the plan parameters like spot shape and spot spacing. The second reason why we believe selecting a d value that corresponds with the transition regions is a better choice is that using a lower d value, resulting in a relative low PBS dose rate, gives a major underestimation of the overall applied dose rate. Third, a practical reason for not using a low d value is that each detector suffers from drift, noise, offset, and latency. These effects have an order of magnitude in the range of the proposed absolute dose threshold of 0.1 Gy. As a result, large variations can be observed in the measured PBS dose rate due to detector limitations.

TRFD, as presented in this work, is based on relative dose accumulation over time, meaning that only the relative PBS dose rate, a percentage of dose per unit time, can be determined. On itself the relative PBS dose rate has limited value, therefore we focused only on the time component Δt, of the PBS dose rate. Incorporating conventional film dosimetry into TRFD to get the absolute dose component ΔD, is a way to go from relative to absolute PBS dose rate. It should be feasible to also perform absolute dose determination with TRFD directly; however, this was considered out of the scope of this study and is subject for future research.

The current study was performed with a continuous proton beam. It would be interesting to know whether the TRFD setup would be applicable to other beam types such as UHDR pulsed electron beams.^18^ These beams consist of microsecond pulses with a repetition rate of tens of milliseconds. Since the response time of Gafchromic film is on the order of microseconds, there is a good chance that it is feasible to measure individual pulses. An important prerequisite for this is the availability of a high‐speed camera with a temporal resolution of 1M fps.

## 5 Conclusion

Time resolved film dosimetry is feasible within clinically relevant limits to perform patient‐specific pre‐treatment verification in pencil‐beam FLASH proton therapy. The current study focused on obtaining the relative PBS dose rate, which can easily be transformed to absolute PBS dose rate by including the absolute dose retrieved from conventional film dosimetry.

## CONFLICT OF INTEREST STATEMENT

HollandPTC reports research collaborations with Varian (Palo, Alto, CA, USA), Siemens Healthineers, Raysearch (Stockholm, Sweden) and DE.TEC.TOR (Turin, Italy). Erasmus MC cancer institute reports research collaborations with Accuray Inc (Sunnyvale, CA, USA), Elekta AB (Stockholm, Sweden), and Varian (Palo, Alto, CA, USA). Leiden University Medical Center reports research collaborations with Varian (Palo, Alto, CA, USA) and Elekta Brachytherapy (Veenendaal, the Netherlands). Spruijt reports chairmanship of machine QA working group within Varian's FLASH Forward Consortium. Godart reports research collaborations with Varian (Palo, Alto, CA, USA). Habraken reports research collaborations with Varian (Palo, Alto, CA, USA), Raysearch (Stockholm, Sweden). Hoogeman reports research collaborations with Varian (Palo, Alto, CA, USA), a test and feedback agreement with Siemens Healthineers, Raysearch (Stockholm, Sweden), and presentation and participation in a ThinkTank meeting for Accuracy Inc (compensation paid to Erasmus MC). Rovituso, Wang and van der Wal report no conflicts of interest. No financial fundings were received to facilitate this study.

## References

[1] Favaudon V , Caplier L , Monceau V , et al. Ultrahigh dose‐rate FLASH irradiation increases the differential response between normal and tumor tissue in mice. Sci Transl Med. 2014;6(245). 10.1126/scitranslmed.3008973 25031268

[2] Montay‐Gruel P , Petersson K , Jaccard M , et al. Irradiation in a flash: unique sparing of memory in mice after whole brain irradiation with dose rates above 100 Gy/s. Radiother Oncol. 2017;124(3):365–369. 10.1016/j.radonc.2017.05.003 28545957

[3] Vozenin M‐C , de Fornel P , Petersson K , et al. The advantage of FLASH radiotherapy confirmed in mini‐pig and cat‐cancer patients. Clin Cancer Res. 2019;25(1):35–42. 10.1158/1078-0432.CCR-17-3375 29875213

[4] MacKay R , Burnet N , Lowe M , et al. FLASH radiotherapy: considerations for multibeam and hypofractionation dose delivery. Radiother Oncol. 2021;164:122–127. 10.1016/j.radonc.2021.09.011 34563608

[5] Daugherty EC , Mascia AE , Sertorio MGB , et al. FAST‐01: results of the first‐in‐human study of proton FLASH radiotherapy. Int J Radiat Oncol Biol Phys. 2022;114(3):s4. 10.1016/j.ijrobp.2022.07.2325

[6] Spruijt K , Mossahebi S , Lin H , et al. Multi‐institutional consensus on machine QA for isochronous cyclotron‐based systems delivering ultra‐high dose rate (FLASH) pencil beam scanning proton therapy in transmission mode. Med Phys. 2024;51(2):786–798. 10.1002/mp.16854 38103260

[7] Yang Y , Shi C , Chen C , et al. A 2D strip ionization chamber array with high spatiotemporal resolution for proton pencil beam scanning FLASH radiotherapy. Med Phys. 2022;49(8):5464–5475. 10.1002/mp.15706 35593052

[8] Folkerts MM , Abel E , Busold S , Perez JR , Krishnamurthi V , Ling CC . A framework for defining FLASH dose rate for pencil beam scanning. Med Phys. 2020;47(12):6396–6404. 10.1002/mp.14456 32910460 PMC7894358

[9] Rahman M , Brůža P , Langen KM , et al. Characterization of a new scintillation imaging system for proton pencil beam dose rate measurements. Phys Med Biol. 2020;65(16);165014. 10.1088/1361-6560/ab9452 32428888 PMC13034663

[10] Levin DS , Friedman PS , Ferretti C , et al. A prototype scintillator real‐time beam monitor for ultra‐high dose rate radiotherapy. Med Phys. 2024. 10.1002/mp.17018 PMC11992679 38456622

[11] Cheung T , Butson MJ , Yu PKN . Post‐irradiation colouration of Gafchromic EBT radiochromic film. Phys Med Biol. 2005;50(20):N281–N285. 10.1088/0031-9155/50/20/N04 16204869

[12] van Battum LJ , Hoffmans D , Piersma H , Heukelom S . Accurate dosimetry with GafChromicTM EBT film of a 6 MV photon beam in water: What level is achievable? Med Phys. 2008;35(2):704–716. 10.1118/1.2828196 18383692

[13] Devic S , Seuntjens J , Hegyi G , et al. Dosimetric properties of improved GafChromic films for seven different digitizers. Med Phys. 2004;31(9):2392–2401. 10.1118/1.1776691 15487718

[14] José Santo R , Habraken SJM , Breedveld S , Hoogeman MS . Pencil‐beam delivery pattern optimization increases dose rate for stereotactic FLASH proton therapy. Int J Radiat Oncol Biol Phys. 2023;115(3):759–767. 10.1016/j.ijrobp.2022.08.053 36057377

[15] Simeonov Y , Weber U , Schuy C , et al. Monte Carlo simulations and dose measurements of 2D range‐modulators for scanned particle therapy. Zeitschrift Für Medizinische Physik. 2021;31(2):203–214. 10.1016/j.zemedi.2020.06.008 32711939

[16] Anderson SE , Grams MP , Wan Chan Tseung H , Furutani KM , Beltran CJ . A linear relationship for the LET‐dependence of Gafchromic EBT3 film in spot‐scanning proton therapy. Phys Med Biol. 2019;64(5):055015. 10.1088/1361-6560/ab0114 30673655

[17] Huang S , Yang Y , Wei S , et al. Implementation of novel measurement‐based patient‐specific QA for pencil beam scanning proton FLASH radiotherapy. Med Phys. 2023;50(7):4533–4545. 10.1002/mp.16458 37198998

[18] Favaudon V , Lentz J‐M , Heinrich S , et al. Time‐resolved dosimetry of pulsed electron beams in very high dose‐rate, FLASH irradiation for radiotherapy preclinical studies. Nucl Instrum Methods Phys Res A. 2019;944:162537. 10.1016/j.nima.2019.162537

